# Occurrence, Antimicrobial Resistance, and Molecular Characterization of *Campylobacter* spp. in Intensive Pig Production in South Africa

**DOI:** 10.3390/pathogens10040439

**Published:** 2021-04-07

**Authors:** Viwe Sithole, Daniel Gyamfi Amoako, Akebe Luther King Abia, Keith Perrett, Linda A. Bester, Sabiha Y. Essack

**Affiliations:** 1 Antimicrobial Research Unit, College of Health Sciences, University of KwaZulu-Natal, Durban 4000, South Africa; viwevisa@gmail.com (V.S.); essacks@ukzn.ac.za (S.Y.E.); 2 Biomedical Resource Unit, College of Health Sciences, University of KwaZulu-Natal, Durban 4000, South Africa; besterl@ukzn.ac.za; 3 Epidemiology Section, KwaZulu-Natal Agriculture & Rural Development-Veterinary Service, Pietermaritzburg 3201, South Africa; keith.perrett@kzndard.gov.za

**Keywords:** molecular epidemiology, *Campylobacter* spp., multidrug resistance, intensive pig production, foodborne pathogens, South Africa, clonality, virulence determinants

## Abstract

*Campylobacter* spp. are among the leading foodborne pathogens, causing campylobacteriosis, a zoonotic infection that results in bacterial gastroenteritis and diarrheal disease in animals and humans. This study investigated the molecular epidemiology of antibiotic-resistant *Campylobacter* spp. isolated across the farm-to-fork-continuum in an intensive pig production system in South Africa. Following ethical approval, samples were collected over sixteen weeks from selected critical points (farm, transport, abattoir, and retail) using a farm-to-fork sampling approach according to WHO-AGISAR guidelines. Overall, 520 samples were investigated for the presence of *Campylobacter* spp., which were putatively identified using selective media with identity and speciation confirmed by polymerase chain reaction (PCR) of specific genes. Resistance profiles were ascertained by the Kirby–Bauer disk diffusion method. Antibiotic resistance and virulence genes were identified using PCR and DNA sequencing. Clonal relatedness was determined using ERIC-PCR. Altogether, 378/520 (72.7%) samples were positive for *Campylobacter* spp., with *Campylobacter coli* being the predominant species (73.3%), followed by *Campylobacter jejuni* (17.7%); 8.9% of the isolates were classified as “other spp”. Relatively high resistance was observed in *C. coli* and *C. jejuni* to erythromycin (89% and 99%), streptomycin (87% and 93%), tetracycline (82% and 96%), ampicillin (69% and 85%), and ciprofloxacin (53% and 67%), respectively. Multidrug resistance (MDR) was noted in 330 of the 378 (87.3%) isolates. The antibiotic resistance genes observed were *tetO* (74.6%), *bla_OXA-_*_61_ (2.9%), and *cmeB* (11.1%), accounting for the resistance to tetracycline and ampicillin. The membrane efflux pump (*cmeB*), conferring resistance to multiple antibiotics, was also detected in most resistant isolates. Chromosomal mutations in *gyrA* (Thr-86-Ile) and *23S rRNA* (A2075G and A2074C) genes, conferring quinolone and erythromycin resistance, respectively, were also found. Of the virulence genes tested, *ciaB*, *dnaJ*, *pldA*, *cdtA*, *cdtB*, *cdtC*, and *cadF* were detected in 48.6%, 61.1%, 17.4%, 67.4%, 19.3%, 51%, and 5% of all *Campylobacter* isolates, respectively. Clonal analysis revealed that isolates along the continuum were highly diverse, with isolates from the same sampling points belonging to the same major ERIC-types. The study showed relatively high resistance to antibiotics commonly used in intensive pig production in South Africa with some evidence, albeit minimal, of transmission across the farm-to-fork continuum. This, together with the virulence profiles present in *Campylobacter* spp., presents a challenge to food safety and a potential risk to human health, necessitating routine surveillance, antibiotic stewardship, and comprehensive biosecurity in intensive pig production.

## 1. Introduction

Antibiotic resistance (ABR) is a global public health crisis. It can be spread through food systems by the movement of livestock and agricultural produce within and between countries, together with human travel [1]. This global rise in antibiotic resistance is attributed to overuse and misuse of antibiotics, incorrectly prescribed antibiotics, and prolonged exposure periods, particularly in food animals. The adoption of intensive animal production to meet the global demand for meat and meat products favors extensive antibiotics used as growth promotors for prophylaxis, metaphylaxis, and food animal treatment. Such uses and long exposure periods create favorable conditions for bacteria to entrench genes that confer drug resistance. The proximity of the animals provides opportunities for the transfer of resistance between the animals on the farm. Most importantly, the resistant bacteria can be transmitted to the human gut microbiota by consuming contaminated food, direct contact with animals, via the environment, and via occupationally exposed workers [1,2].

*Campylobacter* spp. are prevalent in food animals, including pigs, poultry, cattle, goats, and sheep [3,4,5]. Notably, in these animals’ gastrointestinal tract, which serves as a reservoir for the bacteria. These enteric bacterial pathogens are the leading cause of gastroenteritis, specifically campylobacteriosis, a foodborne diarrheal disease of humans and animals. The most frequently encountered *Campylobacter* spp. in foodborne and waterborne infections are *Campylobacter* *jejuni* (*C. jejuni*), *Campalybacter coli* (*C. coli*), and *Campalybacter* *lari* (*C. lari*) [6,7].

Streptomycin, gentamycin, erythromycin, ampicillin, ciprofloxacin, and tetracycline are classified as critically important drugs for food animals by the World Organization for Animal Health (OIE) to protect animal health and welfare, contribute to food safety, and ensure public health [8]. However, there is an associated risk of the development and escalation of antibiotic resistance to these antibiotics should they be excessively used or misused.

Due to these bacteria’s potential to cause foodborne disease outbreaks, comprehensive studies must be carried out to understand the organism’s distribution in food production systems. To achieve this, sampling considerations at different points along the food animal production continuum are of utmost importance, as they provide data on antimicrobial use and resistance and identify transmission (if any) along the food chain. Farms’ samples would indicate resistance arising from on-farm antimicrobial use. Transport and holding pens reflect what is expected to contaminate retail meats and includes cross-contamination with strains persisting in the environment. Post-slaughter addresses meat contamination during in-plant processing, while retail meat products may reflect cross-contamination during handling [9]. Therefore, the current study aimed to describe the molecular epidemiology of antibiotic-resistant *Campylobacter* spp. from farm-to-fork in intensively produced pigs in the uMgungundlovu District KwaZulu-Natal, South Africa, to inform antibiotic use in intensive food animal production and ensure food safety.

## 2. Results

### 2.1. Prevalence of Campylobacter spp.

**Figure 1:**
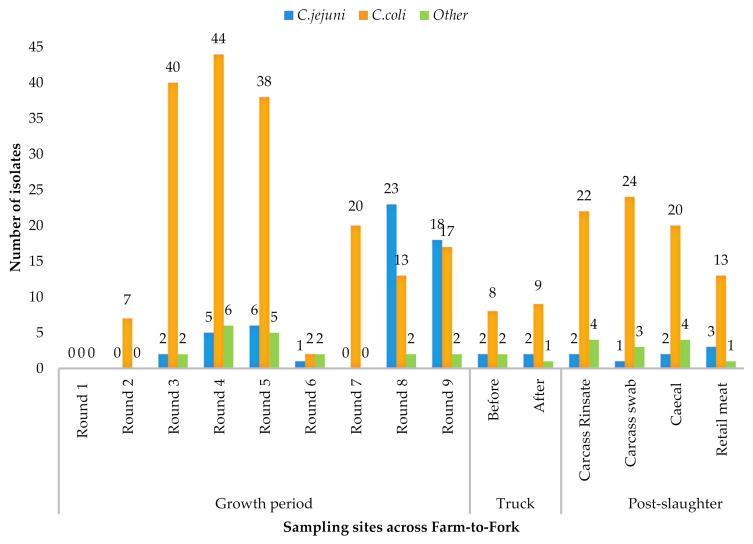
Distribution of *C. coli* and *C. jejuni* spp. along the sampling points of the farm-to-fork continuum.

*Campylobacter* spp. were detected in 378/520 (72.7%) samples across the farm-to-fork continuum, with *C. coli* as the predominant species (*n* = 277, 73.3%), followed by *C. jejuni* (*n* = 67, 17.7%). The remaining isolates were classified as “other *Campylobacter* spp.” *(n =* 34, 8.9%*).* The *Campylobacter* spp. distribution across the farm-to-fork continuum is illustrated in Table 1 and Figure 1. Week-one samples, collected when the piglets were first introduced into the pig pen, and human samples were negative for *Campylobacter* (Figure 1). *C. jejuni* were first isolated on the fourth week of growth and accounted for 21.5% (55/255) of the isolates on the farm. *C. coli* was the predominant species isolated during the transport stage. At retail, 17.6% of the isolates were *C. jejuni*. Other *Campylobacter* species were also isolated at each stage of the food chain (Figure 1).

### 2.2. Antibiotic Susceptibility Profiles

The highest percentage resistance was observed against erythromycin for *C. coli* (89%) and *C. jejuni* (99%); both species recorded the lowest percentage resistance against gentamycin (12% and 11.9%, respectively) (Table 2). The “other” *Campylobacter* species were most resistant to streptomycin (31/34; 91.1%) and least resistant to gentamicin (2/34; 5.8%). 

Multidrug resistance was noted in 330 of the 378 (87.3%) *Campylobacter* isolates tested (Table 3). Categorized by species, 236/277 (85.1%) of *C. coli*, 63/67 (94.0%) of *C. jejuni* and 31/34 (91.1%) of other *Campylobacter* spp. were multidrug-resistant (MDR) (Table 3). A total of 39 antibiograms were observed for *C. coli* and *C. jejuni*. *C. coli* displayed 27 antibiograms compared to *C. jejuni,* which showed 12. The most common MDR profiles identified in the current study in both *C. coli* and *C. jejuni* were resistant to five (AMP-CIP-ERY-TET-STR) and four (AMP-ERY-TET-STR) antibiotics.

### 2.3. Detection of Antibiotic Resistance Genes 

Isolates that showed phenotypic resistance to the selected antibiotics, viz, ciprofloxacin, tetracycline, erythromycin, and ampicillin, were screened for the presence of antibiotic resistance genes. These genes included *gyrA*, *TetO*, *23SrRNA*, *Bla_OXA-_*_61_, and *CmeB*. Most of the tetracycline-resistant isolates (238/319; 74.6%) harbored the *tetO* gene. Only 8/275 (2.9%) of the phenotypically ampicillin-resistant ones carried the *Bla_OXA-_*_61_ gene. The *cmeB* gene, encoding a multidrug efflux pump responsible for resistance to ampicillin, tetracycline, ciprofloxacin, and erythromycin, was detected in 42/378 (11.1%) of the isolates. All 21 *C. coli* and *C. jejuni* isolates tested for the *gyrA* gene exhibited a mutation at the Thr-86-Ile region in the quinolone-resistance-determining region (QRDR) of the *gyrA*. Similarly, the 18 *C. coli* and *C. jejuni* isolates tested for erythromycin resistance exhibited common transitional mutations A2075G and A2074C in the *23S rRNA* gene.

### 2.4. Virulence Genes

**Figure 2:**
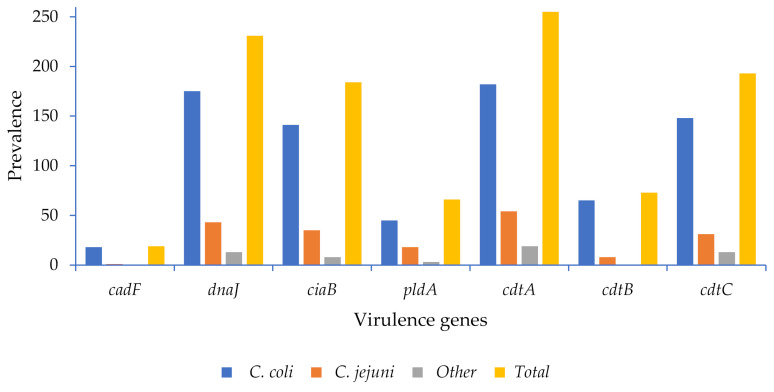
Virulence genes of *Campylobacter* spp. detected in pigs.

The virulence genes investigated in the current study were detected at different frequencies among the species and across the farm-to-fork continuum (Figure 2, Tables S6 and S7). High detection rates were noted for the *cdtA* (67.4%), *dnaJ* (61.1%), *ciaB* (48.6%), and *cdtC* (51%) genes in all isolates, while lower percentages were observed for the *cdtB* (19.3%), *pldA* (17.4%), *cadF* (5%) genes (Figure 2). Both *C. coli* and *C. jejuni* possessed higher frequency of the *cdtA* (66% and 80%), *dnaJ* (63% and 64%), *ciaB* (51% and 52%), and *cdtC* (53.4% and 46.3%) genes, respectively, and lower frequencies of *cdtB*, *pldA*, and *cadF*, respectively, as noted in Figure 2. 

### 2.5. Clonality

**Figure 3:**
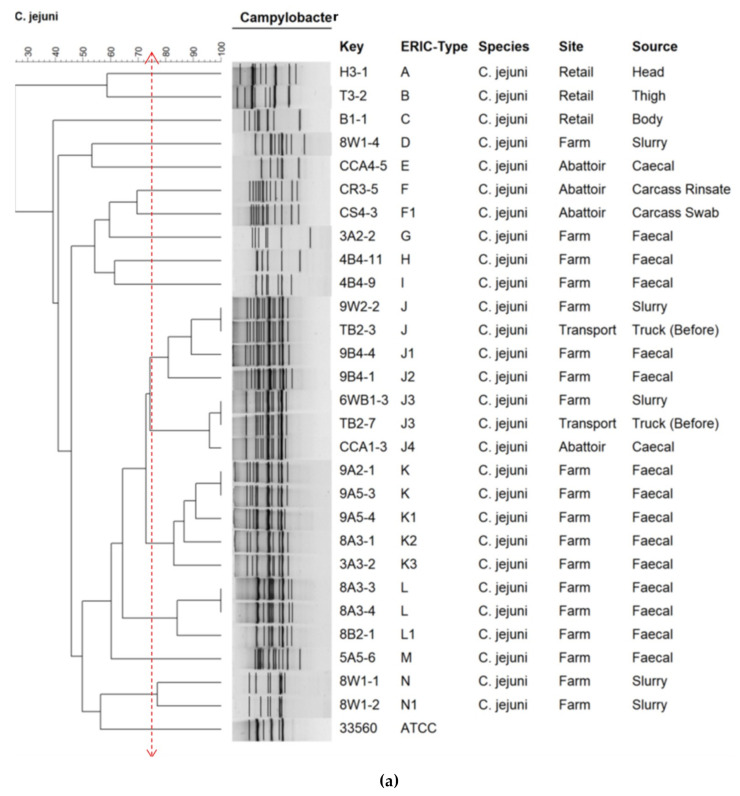

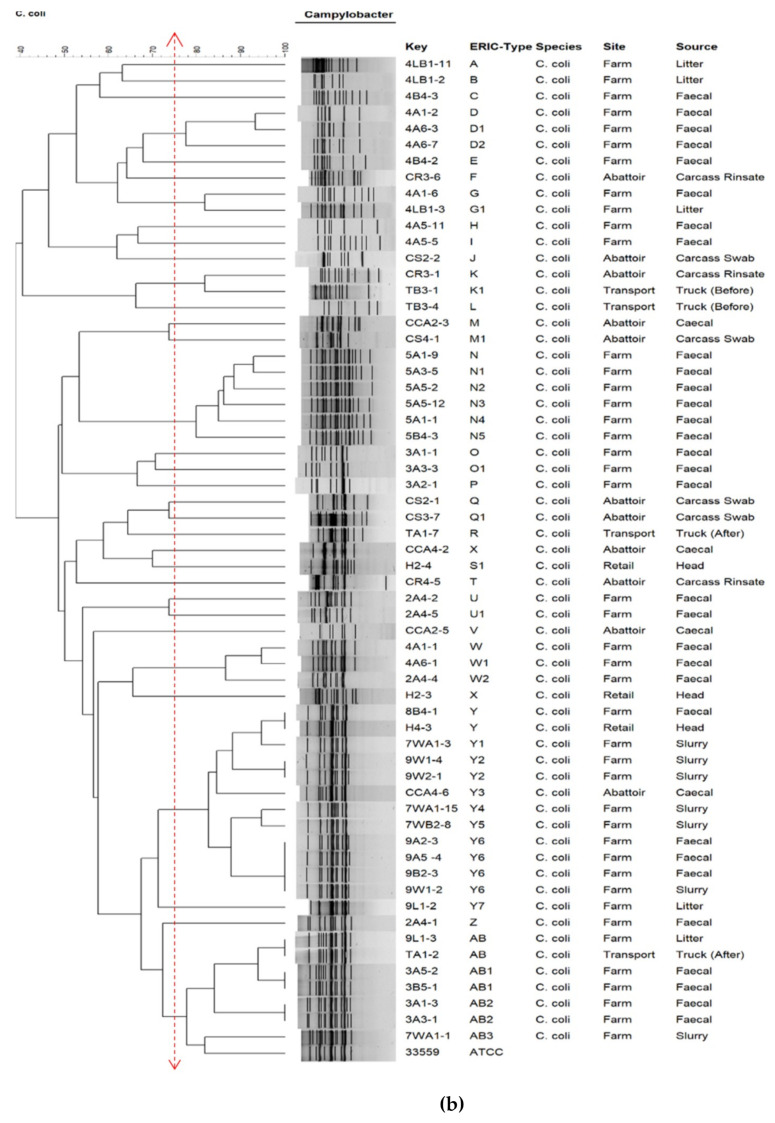
Dendrogram showing ERIC-types of (**a**) *C. jejuni* and (**b**) *C. coli* isolates recovered along the farm-to-fork continuum in relation to the site and source. was used as the control strain. The letters A–Z show the main ERIC-types and subtypes of each isolate. *C. jejuni* ATCC 33560 and *C. coli* ATCC 33559 were used as the control strains.

The dendrogram shows ERIC-types of *C. jejuni* (Figure 3a) and *C. coli* (Figure 3b). Isolates were grouped based on ≥75% similarity. *C. jejuni* was grouped into 14 ERIC-types, designated A–N. It was observed that 54% (15/28) of *C. jejuni* isolates were grouped into three major ERIC-types: J (*n* = 7), K (*n* = 5), and L (*n* = 3). *C. coli* was grouped into 27 ERIC-types designated A–AB. A total of 53% (*n* = 26) *C. coli* fell into three major ERIC-types: Y (*n* = 13), AB (*n* = 7), and N (*n* = 6). The major ERIC clusters were found across the different sites and sources in the farm-to-fork continuum. There was no correlation between the resistance patterns and the ERIC-types.

## 3. Discussion

This study describes the prevalence, antibiotic resistance, virulence profiles, and clonality of *Campylobacter* spp. recovered across the farm-to-fork continuum in an intensive pig production system in the uMgungundlovu District of KwaZulu-Natal, South Africa. The emergence of antibiotic resistance is considered a cross-sectoral problem in the food chain, as antibiotic-resistant bacteria and resistance genes can spread through the food chain to cause infections in humans [10,11]. We showed a *Campylobacter* spp. prevalence of 72.7% (378/520) across the farm-to-fork continuum, with more *C. coli* (73.3%) detected than *C. jejuni* (17.7%). Different levels of resistance and MDR were observed against the different antibiotic classes tested. The different MDR profiles, in different permutations and combinations, attest to the complexity and diversity of resistance across the continuum. Different frequencies of resistance and virulence genes were also observed in the isolates. ERIC-PCR revealed diversity within the isolates, with some evidence of transmission across the farm-to-fork continuum in clone J in *C. jejuni* and clones Y, N, and AB in *C. coli*. These clones contained isolates originating from different sources within the food chain.

### 3.1. Prevalence of Campylobacter spp.

The predominance of *C. coli* in pigs has been previously reported in other studies in South Africa [5], Greece [12], and Nigeria [13]. In the Nigerian study, Gwimi et al. investigated the prevalence of *Campylobacter* spp. in fecal samples of pigs and humans in Zuru Kebi, Nigeria. The study revealed a high percentage (92.66%) of *Campylobacter* spp. in pigs with *C. coli* as the most encountered species at 78.71%, followed by *C. jejuni* at 14.03%, *C. upsaliensis* at 5.40%, and *C. hyointestinalis* as the least encountered at 1.80% [13]. The prevalence and antibiotic resistance of *C. coli* and *C. jejuni* in Greek swine farms showed that 49% of the farms were colonized by *Campylobacter* spp. The predominant species was *C. coli* at 77.4% of the isolates, followed by *C. jejuni* at 22.2% [12]. Igwaran and Okoh (2020) evaluated the prevalence, virulence genes, antimicrobial susceptibility patterns, and resistance gene determinants in *Campylobacter* spp. isolated from retailed meat carcasses in the Eastern Cape Province of South Africa. *Campylobacter* spp. was detected in 28.4% of carcass isolates. *C. coli* had the highest prevalence rate (22.08%), followed by *C. jejuni* (16.66%) and *C. fetus* (3.73%) in all the meat samples. The pork meat samples were colonized with 25.2% of *Campylobacter* spp. [5]. 

### 3.2. Antibiotic Resistance Profiles and Resistance Determinants

The *Campylobacter* spp. isolated in the current study displayed varying percentages of resistance to the antibiotics tested across the farm-to-food continuum (Table 2). Notwithstanding the higher number of farm isolates, resistance was the highest on the farm, where antibiotic exposure was greatest (Tables S4 and S5). The high antibiotic use in intensive food animal production systems contributes significantly to antibiotic resistance among bacteria in the farmed animals [14]. The guidelines for using antimicrobials in the South African pig industry published by the South African Veterinary Association (SAVA) outline the critically and highly important drugs for swine-veterinary and human-swine-veterinary use in South Africa [15]. This policy document lists the use of streptomycin, gentamycin, erythromycin, ampicillin, ciprofloxacin, and tetracycline for pigs, despite their critical and high importance for human medicine. This could explain the resistance observed to these antibiotics in the current study. 

Of note was the significant resistance of *C. coli* and *C. jejuni* isolates to ciprofloxacin (53.4% and 67.1%) compared to nalidixic acid (27.7% and 26.8%), respectively, and their low resistance to gentamycin (12%) (Table 2). Similar findings were reported in a study conducted in Ghana on antibiotic resistance of *Campylobacter* spp. recovered from feces and carcasses of healthy pigs, where high percentage resistance was reported against erythromycin, tetracycline, and ampicillin for *C. coli* and *C. jejuni* isolates. The authors also reported higher resistance to ciprofloxacin than nalidixic acid, as was recorded in our study. Resistance to fluoroquinolones is conferred by the point mutations in the *gyrA* gene, encoding DNA gyrase [16]. The Thr86-Ile substitution in the *gyrA* gene confers cross-resistance to both ciprofloxacin and nalidixic acid; however, mutations of the *gyrA* region in *C. jejuni*, which include Thr86Ala, were reported to be responsible for high-level resistance to nalidixic acid and low-level resistance to ciprofloxacin [17]. Differences in *gyrA* point mutations could explain the observed differences for ciprofloxacin and nalidixic acid resistance. However, more studies need to be conducted to investigate the sequences of *gyrA* and other resistance genes implicated in varying resistance levels to ciprofloxacin and nalidixic acid in *Campylobacter* spp. 

The high resistance to erythromycin, tetracycline, streptomycin, ampicillin, and ciprofloxacin observed in the *C. coli* and *C. jejuni* faecal isolates (Tables S4 and S5) points to a potential high exposer of the isolates to these antibiotics on the farm. Furthermore, the higher percentage resistance of these farm isolates than other sources suggest minimal transmission of these isolates across the farm-to-form continuum. 

A significant number of the resistant isolates in the current study were MDR, with *C.* *coli*, *C. jejuni* and “other” *Campylobacter* spp. recording 85.1%, 94.0%, and 91.1% multidrug resistance, respectively. These findings were similar to those reported in an earlier study conducted in Andhra Pradesh, India [18]. The MDR isolates displayed a total of 39 antibiograms, with *C. coli* displaying more diverse resistance profiles (27 antibiograms) compared to *C. jejuni* (12 antibiograms). The most common MDR profiles in both *C. coli* and *C. jejuni* were resistance to five antibiotics (AMP-CIP-ERY-TET-STR) and resistance to four antibiotics (AMP-ERY-TET-STR), as illustrated in Table 3. Of greater concern was that isolates at the meat processing plant (pig carcasses and final pork products) were also contaminated with antibiotic-resistant *Campylobacter* isolates. The presence of MDR isolates across the farm-to-fork continuum, especially on the final meat portions, necessitating good food preparation hygiene and proper cooking to prevent potential foodborne disease outbreaks. 

Isolates were screened for the presence of the genes conferring resistance to ciprofloxacin, erythromycin, tetracycline, and ampicillin. The common mutations responsible for fluoroquinolone and macrolide resistance include the Thr-86-Ile region in the QRDR and point mutations at positions 2074 and 2075 in the *23SrRNA* [19,20]. These were also detected in all our isolates. The presence of the *Bla_OXA_*_-61_ gene for ampicillin resistance was detected in only 2.9% of our isolates, intimating that other resistance mechanisms to this antibiotic could be involved. An example is the cation-selective MOMP in *C. jejuni* and *C. coli*, which tends to eliminate most β-lactams [21].

The most common mechanism of resistance to tetracycline reported in *Campylobacter* spp. is the protection of the ribosomal binding site by ribosomal protection proteins encoded by the *tetO* gene. This gene was detected in 74.6% of the isolates tested, similar to a study on *Campylobacter* spp. from poultry in KwaZulu-Natal, South Africa, where all isolates resistant to tetracycline carried the *tet(O)* gene [4]. These findings suggest that the *tet(O)* gene appears to be the most common tetracycline resistance determinant in *Campylobacter* spp. from intensive food animal production in KwaZulu-Natal. Nevertheless, the efflux genes, *tetA* and *tetB*, have also been reported [5,22].

Although not all the genes conferring resistance to the tested antibiotics were investigated in this study, the resistance genes detected in the isolates confirmed the phenotypic AST results to a certain extent. Further studies involving a more comprehensive panel of resistance genes should be carried out to better understand the resistance mechanisms in *Campylobacter* in the intensive pig production industry. 

### 3.3. Virulence Determinants

The pathogenicity of *Campylobacter* spp. is mediated by several virulence factors. One of these is the ability to adapt and survive under different temperature changes encountered in the food chain, i.e., exposure to temperatures of 42 °C (chicken intestine), 37 °C (human intestine), and 4 °C (refrigerated foods) [23]. The response to the temperature stress in *Campylobacter* spp. is carried out by several heat shock proteins, including the dnaJ protein encoded by the *dnaJ* gene [24]. Different frequencies of *Campylobacter* virulence-associated genes were observed across the farm-to-fork continuum (Figure 2). The farm had the highest frequency of the genes detected, and this may be because the most significant number of isolates were from the farm; however, a small percentage of the virulence genes was noted in the retail meat products, indicating a pathogenic potential for humans. These included the thermotolerant heat shock gene *dnaJ* and the genes responsible for expressing toxins (*cdtA*, *cdtB*, *cdtC)* (Tables S6 and S7). 

The high frequency of the *dnaJ* gene observed in the present study and its presence in retail meat products indicate a pathogenic potential for humans. The *ciaB* gene (*Campylobacter* invasive antigen B) was also detected in different frequencies throughout the continuum (Tables S6 and S7). This is one of the most important genes responsible for *Campylobacter* invasion, which aids in the translocation of *Campylobacter* into the host cell, thus posing a risk to human health if consumed with contaminated meat products.

The *cdtA*, *cdtB*, and *cdtC* are the three genes needed in a cluster to be functionally active to express the cytotoxins that damage the host nuclear DNA, leading to cell death. *CdtA* and *CdtC* are mainly responsible for host-cell recognition, and the *CdtB* gene is the one that needs to be successfully internalized and delivered into the nucleus of the host cell; its DNaseI-like activity breaks the double-strand DNA leading, to cell death [25,26]. Unlike the *cdtA* and *cdtC* genes, *cdtB* was noted in a smaller percentage. Nevertheless, the detection of these three genes across the continuum raises food safety concerns. 

Similar findings were reported in a study analyzing the prevalence of virulence genes in *Campylobacter* spp. isolated from livestock production systems in South Africa, where all the investigated *Campylobacter* isolates in pigs harbored the genes responsible for adhesion and invasion (*cadF*, *ciaB*, *pldA*), thermotolerance (*dnaJ*), and cytotoxicity (*cdtA*, *cdtB*, and *cdtC*). Contrary to our study, their results showed *cdtA* as the least prevalent gene in *Campylobacter* spp. in pigs and *cdtB*, *cdtC* with high prevalences [27]. It should, however, be noted that not all the virulence genes involved in *Campylobacter* pathogenesis were evaluated in this study, and other genes may contribute to its pathogenesis.

### 3.4. Clonality

The genetic diversity amongst the isolates was investigated using ERIC-PCR. The analysis revealed 14 designated ERIC-types for *C. jejuni* (A–N) and 27 for *C. coli* (A–AB) (Figure 3a,b). Similar results were reported in a study in South Korea on the distribution and molecular characterization of *Campylobacter* spp. at different processing stages in two poultry processing plants [28]. The authors reported that *Campylobacter* clones exhibited high variation and no significant relationships to the species or the processing steps.

However, a single ERIC-type cluster J (subtypes J–J4) was identified in *C. jejuni*, consisting of seven isolates originating from feces, slurry, truck, and caeca (across the continuum). Furthermore, three ERIC-type clusters Y (subtypes Y–Y7), consisting of 13 isolates originating from feces, slurry, litter, caeca, and retail meat, AB (subtypes AB–AB3) consisting of 7 isolates originating from feces, litter, slurry, and truck samples, and N (subtypes N–N5), consisting of six isolates originating from feces, litter, slurry, caeca, and retail meat samples were elucidated in *C. coli*. These results suggest that there could be transmission along the food chain, with similar clones found across the continuum, specifically in retailed meat. This needs urgent mitigating measures to curb this potential food safety threat. Of note, although ERIC-PCR has a shorter turnaround time, it is less discriminatory. Therefore, further studies involving more resolute typing approaches, such as whole-genome sequencing, are recommended. 

## 4. Materials and Methods

### 4.1. Ethical Considerations

Ethical approval for this study was received from the Animal Research Ethics Committee (Reference: AREC 007/018) and the Biomedical Research Ethics Committee (Reference: BCA444/16) of the University of KwaZulu-Natal. We also obtained Section 20A permission to conduct the study from the South African National Department of Agriculture, Forestry and Fisheries (Reference No.: 12/11/1/5). All information obtained from the farm was kept confidential as part of the memorandum of understanding (MOU) between the Antimicrobial Research Unit and the farm. 

### 4.2. Study Site

The study was carried out at an intensive pig production facility in the uMgungundlovu District, KwaZulu-Natal (KZN), South Africa. 

### 4.3. Participants

Hand and nasal samples were obtained from occupationally exposed adult (>18 years old) workers on the farm upon explicit, voluntary, written informed consent.

### 4.4. Sampling 

The “farm-to-fork approach” recommended by WHO-AGISAR (Advisory Group on Integrated Surveillance of Antimicrobial Resistance) was implemented in this study [9]. 

Samples were collected across the farm-to-fork continuum as follows:

- Production (animals on the farm): samples collected included feces, litter (the bedding), and slurry. These were collected every two weeks (nine rounds of sampling) from birth to slaughter over four months (September 2018–January 2019). The block sampling method was used to ensure an even representation of the entire herd within the pig pen house.
- Hand and nasal samples were collected from the farmworkers on Weeks 3, 5–7, and 9 over four months based on individual voluntary consent.
- Holding and transport samples consisted of swab samples from the holding pens and truck floor. These were collected before and after the transportation of the pigs to the abattoir.
- Post-slaughter (abattoir) samples included carcass swabs, caecal samples, and carcass rinsate.
- Swab samples of meat products, including whole cuts (head, body, and thigh) sold to consumers, were collected from the meat processing plant at the retail point.

The final sample size across all sources was 520 (Figure S1).

### 4.5. Isolation of Campylobacter spp.

Samples were processed as previously described [29,30] with slight modifications. Briefly, 1 g of each fecal and litter sample was suspended in 4 mL of enrichment blood-free *Campylobacter* charcoal broth (Himedia Laboratories Pvt., Ltd., Mumbai, India) supplemented with modified charcoal cefoperazone deoxycholate (mCCD) *Campylobacter* selective supplements. Ten swab samples were pooled into 1 mL sterile distilled water and 4 mL of *Campylobacter* charcoal broth. Following incubation at 42 °C for 24 h under microaerophilic conditions, approximately six drops (100 µL) of the enrichment charcoal broth were filtered through a 0.45 µm pore size cellulose nitrate filter (Sartorius Stedim Biotech, Gottingen, Germany) onto mCCDA *Campylobacter* blood-free selective agar base (Oxoid LTD, Basingstoke, United Kingdom) containing CCDA selective supplement SRO 155E (Oxoid LTD, Basingstoke, Hampshire, England) and incubated at 42 °C for 24 h under microaerophilic conditions [30]. Ten single colonies showing typical morphology of *Campylobacter* spp. with smooth, colorless translucent to grey appearance were randomly selected from the selective agar plates and subcultured onto Tryptose blood agar base (Biolab, Longmeadow Business Estate South, Modderfontein, South Africa) supplemented with 5% defibrinated sheep blood. Single colonies were stored in tryptone soya broth (Oxoid LTD, Basingstoke, Hampshire, England) with 20% glycerol at −80 °C for further investigations [29]. Quality control strains *C. jejuni* ATCC 33560 and *C. coli* ATCC 33559 were used in the bacterial identification process. 

### 4.6. DNA Extraction

DNA was extracted using the conventional boiling method as previously described [29,30] with slight modifications. Briefly, colonies were suspended in 200 µL sterile distilled water, vortexed to homogenize cells, boiled at 100 °C for 15 min, then cooled on ice for 5 min. The suspension was then centrifuged at 13,000 rpm for 5 min, and the supernatant collected and stored at −25 °C for further use. The *C. jejuni* ATCC 33560 and *Campylobacter coli* ATCC 33559 controls were subjected to the same DNA isolation process. The concentration and purity of the DNA were ascertained using a Nanodrop 2000 UV-Vis Spectrophotometer (Thermo-Fisher Scientific, Waltham, MA, USA).

### 4.7. Molecular Confirmation of Isolates

*Campylobacter* isolates were confirmed to genus and species level using real-time polymerase chain reaction (RT-PCR). The genus and species-specific primers used for amplification (Table S1) were purchased from Inqaba Biotechnical Industries (Pty) Ltd. Pretoria, South Africa. The *Campylobacter* genus-specific *16SrRNA* gene was used to confirm isolates to genus level, and species identification focused on two species-specific genes, viz., the hippuricase gene (*hipO)* gene, specific for *C. jejuni*, and the aspartokinase gene *(asp*) gene, specific for *C. coli* (Table S1). *C. jejuni* ATCC 33560 and *C. Coli* ATCC 33559 served as positive controls, while a reaction mixture without template DNA was used as a negative control. 

The reaction was carried out in a total volume of 10 µL consisting of 5 µL of a 2× Luna^®^ Universal qPCR master mix (Biolabs, New England Ipswich, MA, USA), 0.5 µL forward and reverse primer mixture, 3 µL sample DNA, and 1 µL of nuclease-free water. The following cycling conditions were used as previously optimized by [31] with slight modifications: initial activation stage at 95 °C for 10 min, followed by 40 cycles of denaturation at 95 °C for 10 s, specific annealing temperature for each primer (Table S1) for 15 s, an extension at 72 °C for 20 s, and a final extension at 72 °C for 5 min. A melt curve was achieved by increasing the temperature from 60 °C to 95 °C [4,31]. All reactions were performed on a QuantStudio 5^TM^ Real-Time PCR System (ThermoFisher Scientific, Waltham, MA, USA), and the melt curve analysis was carried out using QuantStudio Design & Analysis software version 1.4.3 (ThermoFisher Scientific, Waltham, MA, USA).

### 4.8. Antibiotic Susceptibility Testing

Antibiotic susceptibility testing (AST) was performed using the Kirby–Bauer disk diffusion method on Mueller Hinton Agar that was supplemented with 5% horse blood as recommended by the European Committee on Antimicrobial Susceptibility Testing (EUCAST) and Clinical and Laboratory Standard Institute guidelines (CLSI) [32,33]. The seven antibiotics that were tested with their corresponding concentrations were gentamicin (10 µg), streptomycin (10 µg), erythromycin (15 µg), ampicillin (10 µg), ciprofloxacin (5 µg), nalidixic acid (30 µg), and tetracycline (30 µg), as recommended by the WHO-AGISAR guidelines [9]. AST results for ciprofloxacin, erythromycin and tetracycline were interpreted using EUCAST breakpoints, while those for ampicillin, gentamycin, streptomycin and nalidixic acid were interpreted using CLSI guidelines. 

Briefly, a sterile swab was used to remove about two to three colonies from a 48-h culture into 250 mL of sterile distilled water to obtain an inoculum equivalent to 0.5 McFarland standard as recommended by the EUCAST guideline. The suspension was inoculated onto the Mueller Hinton agar (Biolab, Longmeadow Business Estate South, Modderfontein, South Africa). This was followed by dispensing antibiotics discs onto the agar plates using a multipoint disc inoculator. Plates were then incubated under microaerophilic conditions in an anaerobic jar created by CampyGen (Oxoid LTD, Basingstoke UK) at 42 °C for 24 h and reincubated if there was insufficient growth [4]. Results were interpreted by measuring zones of inhibition around each antibiotic disc in millimeters. Zones were recorded and interpreted following EUCAST and CLSI breakpoints. Isolates displaying resistance to one or more antibiotics from three or more distinct antibiotic classes were classified as multidrug-resistant (MDR) isolates. *C. jejuni* ATCC 33560, *C. coli* ATCC33559 were used as control strains [32,33].

### 4.9. Detection of Antibiotic Resistance and Virulence Genes 

Isolates were tested for the presence of selected resistance and virulence genes using Real-time PCR (RT-PCR), the former informed based on AST results. The primers used and PCR conditions are as listed in Tables S2 and S3, respectively. Primers were purchased from Inqaba Biotechnical Industries (Pty) Ltd., Pretoria, South Africa. 

All the isolates phenotypically resistant to ciprofloxacin, tetracycline, erythromycin, and ampicillin were screened for the presence of selected resistance genes, viz., *gyrA*, *TetO*, *23SrRNA*, *bla_OXA-_*_61_, and *CmeB*. These are the most frequently reported antibiotic resistance genes in *Campylobacter* spp. in the literature. Selected PCR products were sequenced to identify known/novel mutations conferring resistance on an ABI 3130XL Genetic Analyser using the Sanger method of DNA sequencing by Inqaba Biotechnical Industries (Pty) Ltd., Pretoria, South Africa. The sequences were analyzed using Basic Local Alignment Search Tool^®^ 2.0 software, available from the National Center for Biotechnology Information. Results were compared to other known similar *Campylobacter* gene sequences in GENBANK. 

Seven virulence genes involved in adhesion (*cadF*, *pldA*), thermotolerance (*dnaJ*), invasion (*ciaB*), and toxin production (*cdtA*, *cdtB and cdtC*) were investigated using primers and PCR conditions described in Table S3. 

The reaction was carried out in a total volume of 10 µL made up of 5 µL of a 2× Luna^®^ Universal qPCR master mix (New England Biolabs, Ipswich, MA, USA), 0.5 µL of each forward and reverse primer, 3 µL of template DNA and 1 µL of nuclease-free water. The already optimized PCR cycling conditions [4,31] were followed. *C. jejuni* ATCC 29428 served as a positive control for the virulence genes tested. *C. jejuni* 33560 was used as a positive control for the resistance genes *tetO*, *bla_OXA-_*_61_, *23S rRNA* at position 2074 and 2075, while *C. coli* 33559 was used as a positive control for the *gyrA* and *cmeB* genes. All reactions were performed on a QuantStudio^TM^ 5 Real-Time PCR System (ThermoFisher Scientific, Waltham, MA, USA), and melt curve analysis was carried out using QuantStudio Design & Analysis software version 1.4.3 (ThermoFisher Scientific, Waltham, MA, USA).

### 4.10. Clonality (ERIC-PCR)

The clonality of the isolates was determined by the Enterobacterial Repetitive Intergenic Consensus (ERIC)-PCR using ERIC1R (5′-ATG TAA GCT CCT GGG GAT TCA C-3′) and ERIC2 (5′-AAG TAA GTG ACT GGG GTG AGC G-3′) primers as described by [34]. Isolates for this analysis were selected based on source and antibiograms. Briefly, isolates with antibiograms showing similar resistance patterns from different sources along the farm-to-fork continuum were selected to determine clonal relatedness between the isolates. The reaction was carried out in a total volume of 20 µL consisting of 12.5 µL of DreamTaq Green PCR Master Mix (2X) (ThermoFisher Scientific, Vilnius, Lithuania), 0.1 µL of each forward and reverse primer (final concentration of 10 μM), 3 µL of template DNA and 4.3 µL of nuclease-free water. The PCR amplification conditions were as previously described [34]. The amplified products were electrophoresed on a 1% agarose gel at 75 V for 3 h in a 1 X Tris-acetate-EDTA (TAE) buffer (Bioconcept Ltd., Allschwil, Switzerland). A 100 bp DNA ladder (New England Biolabs, Hitchin, Hertfordshire, UK) was used as the molecular weight marker. The gel images were captured using a Gel Doc™ XR+ imaging system (Bio-Rad Laboratories, Inc. Hercules, California, USA). Fingerprint patterns were analyzed using SYNGENE Bionumerics software version 6.6 (Applied Maths NV, Sint-Martens-Latem, Belgium). A band tolerance of 10% was used for inputting gel images. Cluster generation used Pearson correlation with a 1% optimization and an unweighted pair group with arithmetic averages (UPGMA) to create dendrograms. Clusters were determined using a 75% similarity cut-off [35]. 

### 4.11. Data Analysis and Interpretation

The data were analyzed using Microsoft Excel 2018 and Statistical Package for Social Sciences (SPSS) version 23 (IBM Corporation, Armonk, New York, NY, USA). Descriptive statistics were used to describe the occurrence of *Campylobacter* isolates, phenotypic resistance profiles, and genotypic profiles from different sources.

## 5. Conclusions

We found a high prevalence of multidrug-resistant *Campylobacter* spp., with some evidence, albeit minimal, of transmission across the farm-to-fork continuum, presenting a potential risk to human health. The antibiotic resistance and virulence profiles exacerbate the risk of foodborne infection, further exacerbated by the reduction in antibiotic treatment options. This calls for enhanced antibiotic stewardship, comprehensive biosecurity, and good animal husbandry in intensive pig production together with the implementation of routine surveillance, ideally at a genomic level to understand the trends and molecular epidemiology of antibiotic-resistant *Campylobacter* spp. across the farm-to-fork continuum. 

## Figures and Tables

**Table 1:** Prevalence of *Campylobacter* spp. across the farm to fork continuum.

Source	Total Samples	No. of Positive Samples	Sample Proportion Among the Positives	Total Isolates	*C. coli* (% Per Site)	*C. jejuni* (% Per Site)	Other (% Per Site)
Feces	232	195	51.50%	255	181 (65.3)	55 (82.1)	19 (59.9)
Litter	16	16	4.20%				
Slurry	97	44	11.60%				
Truck and crate swabs	40	24	6.30%	24	17 (6.1)	4 (5.9)	3 (8.8)
Carcass swabs	39	31	8.20%	82	66 (23.8)	5 (7.5)	11 (32.4)
Carcass rinsates	32	25	6.60%				
Caeca	44	26	6.80%				
head, body and Thigh	20	17	4.40%	17	13 (4.7)	3 (4.5)	1 (2.9)
TOTAL	520	378		378	277	67	34

**Table 2:** Antibiotic resistance (%) of *Campylobacter* spp. isolated from pigs.

Species and Number (%) of Resistant Isolates					
Antimicrobial Class	Antimicrobials	*C. coli* (*n* = 277)	*C. jejuni* (*n* = 67)	Others (*n* = 34)	Total (*n* = 378)
Aminoglycosides	Gentamycin	34 (12.2)	8 (11.9)	2 (5.8)	44 (11.6)
	Streptomycin	240 (86.6)	62 (92.5)	31 (91.1)	333 (88.0)
Macrolides	Erythromycin	247 (89.1)	66 (98.5)	30 (88.2)	343 (90.7)
Penicillins	Ampicillin	191 (68.9)	57 (85.0)	28 (82.3)	276 (73.0)
Quinolones	Ciprofloxacin	148 (53.4)	45 (67.1)	23 (67.6)	216 (57.1)
	Nalidixic acid	77 (27.7)	18 (26.8)	8 (23.5)	103 (27.2)
Tetracyclines	Tetracycline	227 (81.9)	64 (95.5)	28 (82.3)	319 (84.3)

**Table 3:** Antibiograms of *Campylobacter* spp. isolated from pigs.

No. of Resistance	Resistance Patterns	Number of Isolates Per Sample Type								*C. coli* (*n* = 277)	*C. jejuni* (*n* = 67)	Other (*n* = 34)	Total (*n* = 378)
		F	S	L	TC	CR	CS	C	RM				
7	AMP+NAL+CIP+ERY+TET+GEN+STR	14	2							10	5	1	16
	TOTAL									10	5	1	16
6	AMP+NAL+CIP+ERY+TET+STR	16	3	1		3	1		1	17	7	1	25
	NAL+CIP+ERY+TET+GEN+STR	2	2		1					5	0	0	5
	AMP+CIP+ERY+TET+GEN+STR	13	1							11	2	1	14
	AMP+NAL+ERY+TET+GEN+STR	1								1	0	0	1
	TOTAL									34	9	2	45
5	AMP+CIP+ERY+TET+STR	55	9	4	4	1	2	15	1	56	25	13	94
	AMP+NAL+ERY+TET+STR	5					1			4	1	1	6
	AMP+NAL+CIP+ERY+STR					2	2			4	0	0	4
	NAL+CIP+ERY+TET+STR	2	1							1	2	0	3
	NAL+CIP+TET+GEN+STR		1							1	0	0	1
	TOTAL									66	28	14	108
4	ERY+TET+GEN+STR	2								2	0	0	2
	AMP+ERY+TET+STR	37	7		17	4	1	8	1	51	15	6	72
	CIP+ERY+TET+STR	2		2			1		5	9	0	1	10
	AMP+CIP+ERY+STR					5	7			9	1	2	12
	NAL+ERY+TET+STR			1						1	0	0	1
	NAL+CIP+ERY+TET	2		1		1				3	1	0	4
	AMP+NAL+ERY+TET	2								1	1	0	2
	NAL+CIP+ERY+STR						1			0	0	1	1
	AMP+NAL+ERY+STR					5	6			10	0	1	11
	NAL+CIP+TET+STR	1	11	1						11	0	2	13
	TOTAL									97	18	13	128
3	AMP+ERY+TET	2			1					2	0	1	3
	NAL+ERY+TET	1								1	0	0	1
	ERY+TET+STR	9		2						9	2	0	11
	AMP+ERY+STR	1				3	7		1	12	0	0	12
	CIP+ERY+STR								2	1	1	0	2
	AMP+CIP+ERY							1		1	0	0	1
	ERY+TET+GEN	1								1	0	0	1
	CIP+ERY+TET							1	1	2	0	0	2
	TOTAL									29	3	1	33
	Total MDR isolates									236	63	31	330
	Percentage MDR									85.1%	94.0%	91.1%	
	None MDR									41	4	3	48

AMP = ampicillin, ERY = erythromycin, CIP = ciprofloxacin, GEN = gentamicin, STR = streptomycin, TET = tetracycline, NAL = nalidixic acid, F = feces, S = slurry, L = litter, TC = transport crates, CR = carcass rinsate, CS = carcass swab, RM = retail meat.

## Data Availability

All data is contained within this article. Additional data is available in Supplementary Materials.

## Supplementary Materials

The following are available online at https://www.mdpi.com/article/10.3390/pathogens10040439/s1, Figure S1: Sampling framework, Table S1: Primers used for confirmation of *Campylobacter* to genus and species level, Table S2: Primers used for the detection of antibiotic resistance genes, Table S3: Primers used for the detection of virulence genes, Table S4: Antibiotic resistance (%) of *C. coli* across the farm-to-fork continuum, Table S5: Antibiotic resistance (%) of *C. jejuni* across the farm-to-fork continuum, Table S6: Prevalence (%) of virulence genes detected in *C. coli* (*n* = 277) across the farm-to-fork continuum, Table S7: Prevalence (%) of virulence genes detected in *C. jejuni* (*n* = 67) across the farm-to-fork continuum.
