# Supplementary material for: Occurrence, Antimicrobial Resistance, and Molecular Characterization of Campylobacter spp. in Intensive Pig Production in South Africa
Source: Pathogens. 2021 Apr 7;10(4):439. doi: 10.3390/pathogens10040439 (PMC8067824; doi:10.3390/pathogens10040439)
Supplement: Supplementary file 1 [file pathogens-10-00439-s001.pdf]

Supplementary Material

Occurrence, Antimicrobial Resistance, and Molecular Characterisation of *Campylobacter* Spp. in Intensive Pig Production in South Africa

Viwe Sithole [1], Daniel Gyamfi Amoako [1,2*], Akebe Luther King Abia [1*], Keith Perrett [3], Linda A. Bester [2] and Sabiha Y. Essack [1]

[1] Antimicrobial Research Unit, College of Health Sciences, University of KwaZulu-Natal, Durban 4000, South Africa; viwevisa@gmail.com (V.S.); amoakodg@gmail.com (D.G.A.); lutherkinga@yahoo.fr (A.L.K.A.); essacks@ukzn.ac.za (S.Y.E.)

[2] Biomedical Resource Unit, College of Health Sciences, University of KwaZulu-Natal, Durban 4000, South Africa; besterl@ukzn.ac.za (L.A.B.)

[3] Epidemiology Section, KwaZulu-Natal Agriculture & Rural Development-Veterinary Service, Pietermaritzburg 3201, South Africa; keith.perrett@kzndard.gov.za (K.P.)

* Correspondence: amoakodg@gmail.com (D.G.A.), Tel.: +27(0)843308957; ORCID: 000-0003-3551-3458; lutherkinga@yahoo.fr (A.L.K.A.), Tel.: +27(0)734403343, ORCID: 000-0002-5194-2810

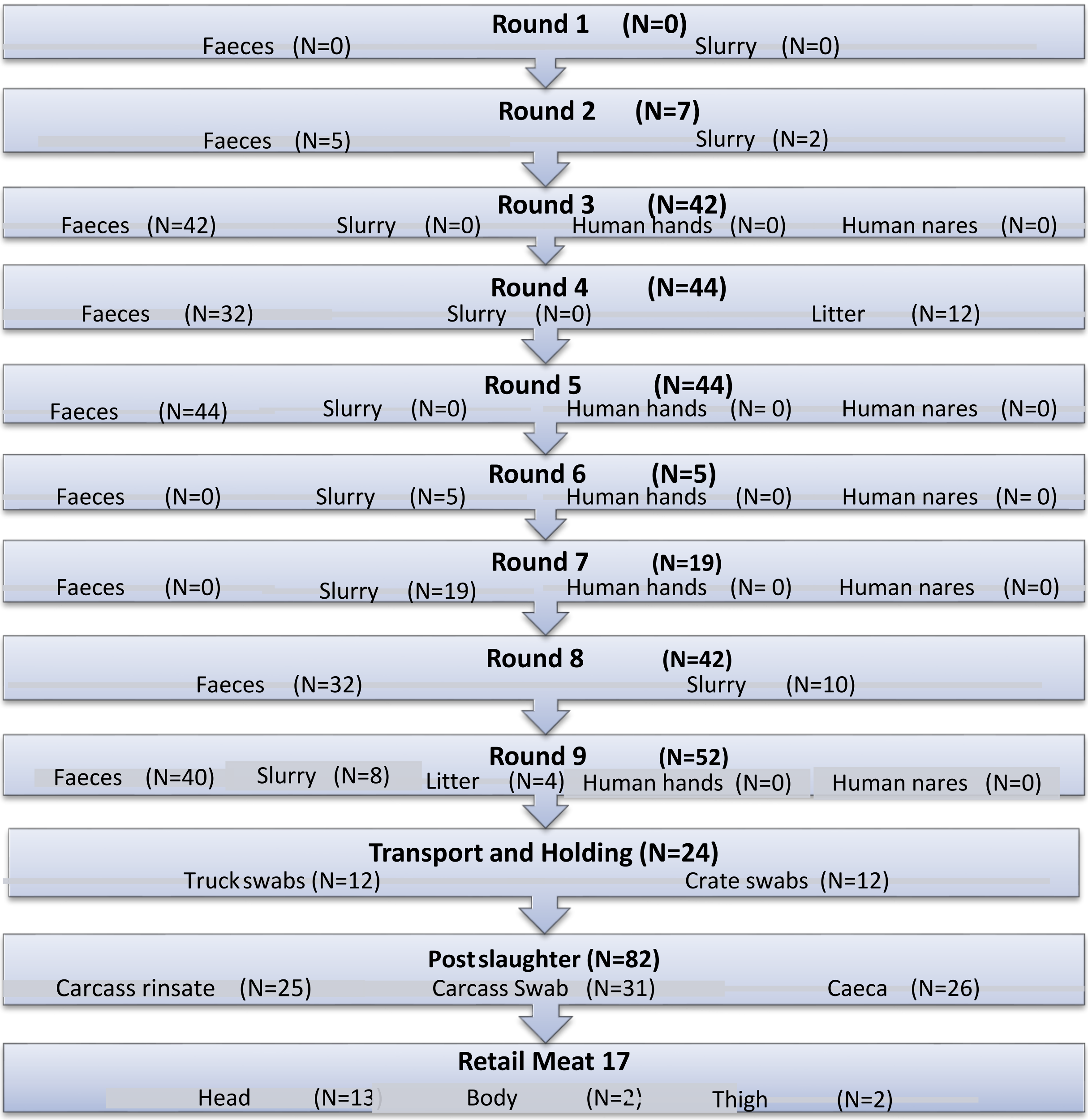

Figure S1: Sampling framework. "N" denotes the number of confirmed *Campylobacter spp.* at each sampling point and source.

Table S1: Primers used for confirmation of *Campylobacter* to genus and species level.

**Target gene**	**Primer Sequence (5' – 3')**	**Product Size (bp)**	**Annealing Temperature (℃)**	**References**
Asp	F-GGTATGATTTCTACAAAGCGAG<br>R-ATAAAAGACTATCGTCGCGTG	500	60	[4]
hipO	F-GAAGAGGGTTTGGGTGGTG<br>R-AGCTAGCTTCGCATAATAACTTG	735	66	[4]
16S rRNA	F-GGATGACACTTTTCGGAGC<br>R-CATTGTAGCACGTGTGTC	816	58	[4]

Table S2: Primers used for the detection of antibiotic resistance genes.

Target gene	Primer Sequence (5’ – 3’)	Product Size (bp)	Annealing Temperature(℃)	References
23S rRNA at position 2074	F-TTAGCTAATGTTGCCCGTACCG<br>R-AGTAAAGGTCCACGGGGTCTCG	485	59	[29]
23S rRNA at position 2075	F-TTAGCTAATGTTGCCCGTACCG<br>R-TAGTAAAGGTCCACGGGGTCGC	486	59	[29]
gyrA (C. coli)	F-GAAGAATTTTATATGCTATG<br>R-TCAGTATAAC GCATCGCAGC	235	53	[4]
gyrA (C. jejeuni)	F-ACGCAAGAGAGATGGTT<br>R-TCAGTATAACGCATCGCAGC	270	45	[4]
cmeB	F-GACGTAATGAAGGAGAGCCA<br>R-CTGATCCACTCCAGCTATG	1166	50	[29]
tet(O)	F-GGCGTTTTGTTTATGTGCG<br>R-ATGGACAACCCGACAGAAGC	559	49	[4]
blaOXA$_{-61}$	F-AGAGTATAATACAAGCG<br>R-TAGTGAGTTGTCAAGCC	372	54	[4]

Table S3: Primers used for the detection of virulence genes.

Target gene	Primer Sequence (5' – 3')	Product Size (bp)	Annealing Temperature (°C)	References
cadF	F-TTGAAGGTAATTTAGATATG<br>R-CTAATACCTAAAGTTGAAAC	400	43	[30]
ciaB	F-TGCGAGATTTTTCGAGAATG<br>R-TGCCCGCCTTAGAACTTACA	527	54	
dnaJ	F-ATTGATTTTGCTGCGGGTAG<br>R-ATCCGCAAAAGCTTCAAAAA	177	50	
pldA	F-AAGAGTGAGGCGAAATTCCA<br>R-GCAAGATGGCAGGATTATCA	385	46	
cdtA	F-CCTTGTGATGCAAGCAATC<br>R-ACACTCCATTTGCTTTCTG	370	49	
cdtB	F-GTTAAAATCCCCTGCTATCAACCA<br>R-GTTGGCACTTGGAATTTGCAAGGC	495	51	
cdtC	F-CGATGAGTTAAAACAAAAAGATA<br>R-TTGGCATTATAGAAAATACAGTT	182	48	

Table S4: Antibiotic resistance (%) of *C. coli* (*n* = 277) across the farm-to-fork continuum.

	Farm			Transport	Abattoir			Retail
**Antibiotic Panel**	**Faeces**	**Litter**	**Slurry**	**Truck/ Crates**	**Carcass Swab**	**Carcass Rinsate**	**Caecal**	**Retail Meat**
Ampicillin	34%	2%	4%	5%	8%	8%	7%	1%
Nalidixic acid	13%	1%	6%	0%	3%	4%	0%	0%
Ciprofloxacin	26%	3%	8%	2%	3%	4%	5%	3%
Erythromycin	46%	5%	6%	6%	8%	8%	7%	4%
Tetracycline	45%	6%	10%	6%	1%	3%	7%	3%
Gentamycin	10%	0%	2%	0%	0%	0%	0%	0%
Streptomycin	40%	4%	9%	6%	8%	8%	7%	4%

Table S5: Antibiotic resistance (%) of *C. jejuni* (*n* = 67) across the farm-to-fork continuum.

	Farm			Transport	Abattoir			Retail
**Antibiotic Panel**	**Faeces**	**Litter**	**Slurry**	**Truck/ Crates**	**Carcass Swab**	**Carcass Rinsate**	**Caecal**	**Retail Meat**
Ampicillin	57%	0%	13%	6%	0%	2%	3%	3%
Nalidixic acid	16%	0%	8%	0%	2%	2%	0%	0%
Ciprofloxacin	43%	0%	13%	0%	2%	3%	2%	5%
Erythromycin	67%	0%	13%	6%	2%	3%	3%	5%
Tetracycline	67%	0%	13%	6%	2%	2%	3%	3%
Gentamycin	8%	0%	3%	0%	2%	0%	0%	0%
Streptomycin	63%	0%	13%	6%	2%	2%	3%	5%

Table S6: Prevalence (%) of virulence genes detected in *C. coli* (*n* = 277) across the farm-to-fork continuum

**Virulence genes**	**Farm**			**Transport**	**Abattoir**			**Retail**
	**Faeces**	**Litter**	**Slurry**	**Truck and Crates**	**Carcass Swab**	**Carcass Rinsate**	**Ceacal**	**Retail Meat**
cadF	2%	0%	3%	0%	1%	1%	0%	0%
dnaJ	26%	6%	8%	3%	7%	8%	1%	4%
ciaB	23%	2%	6%	3%	7%	4%	4%	2%
pldA	11%	3%	1%	0%	0%	0%	0%	1%
cdtA	32%	6%	7%	3%	7%	6%	3%	1%
cdtB	17%	1%	0%	1%	1%	1%	1%	1%
cdtC	21%	2%	5%	5%	8%	5%	5%	2%

Table S7: Prevalence (%) of virulence genes detected in *C. jejuni* (n = 67) across the farm-to-fork continuum.

**Virulence genes**	**Farm**			**Transport**	**Abattoir**			**Retail**
	**Faeces**	**Litter**	**Slurry**	**Transport Crates**	**Carcass Swab**	**Carcass Rinsate**	**Ceacal**	**Retail Meat**
cadF	0%	0%	0%	0%	1%	0%	0%	0%
dnaJ	51%	0%	7%	1%	1%	0%	0%	3%
ciaB	37%	0%	6%	3%	1%	1%	1%	0%
pldA	22%	0%	4%	0%	0%	0%	0%	0%
cdtA	58%	0%	13%	3%	1%	1%	3%	0%
cdtB	7%	0%	1%	0%	1%	0%	0%	1%
cdtC	27%	0%	7%	3%	1%	1%	3%	3%
